# Radiology–pathology correlation of endometrial carcinoma assessment on magnetic resonance imaging

**DOI:** 10.1186/s13244-022-01218-3

**Published:** 2022-04-25

**Authors:** Eveline Dokter, Lyndal Anderson, Soo-Min Cho, Violette Cohen-Hallaleh, Kim May Lam, Samir A. Saidi, Yu Xuan Kitzing

**Affiliations:** 1 Department of Radiology, Royal Prince Alfred Hospital, Missenden Road, Camperdown, NSW 2050 Australia; 2 Department of Radiology, Leiden University Medical Center, Albinusdreef 2, 2333 ZA Leiden, The Netherlands; 3 Department of Tissue Pathology and Diagnostic Oncology, Royal Prince Alfred Hospital, Missenden Road, Camperdown, NSW 2050 Australia; 4 Sydney Medical School, University of Sydney, Sydney, Australia; 5 Central Clinical School, University of Sydney, Sydney, Australia; 6 Lifehouse Gynaecological Oncology Group, Chris O’Brien Lifehouse, Sydney, Australia

**Keywords:** Endometrial carcinoma, Magnetic resonance imaging, FIGO staging, Radiology-pathology correlation

## Abstract

Endometrial carcinoma is the most common gynaecological cancer in developed countries. Most cases are low-volume/low-grade tumour at presentation; however, high-grade subtypes may present with locally advanced disease with higher propensity for spread outside of the pelvis. MRI has a role in local staging of the tumour and helping the clinicians in treatment decision making. This pictorial essay gives examples of endometrial carcinoma at different stages with histological correlation. It also explores the potential limitations and pitfalls of imaging in this context.

## Key points

- MRI plays an important role in staging endometrial carcinoma particularly in the evaluation of depth of invasion into the myometrium.
- Factors such as large intracavitary tumour, cornual tumour location, variable tumour appearance, adenomyosis and fibroid can affect the MRI accuracy in assessing the depth of myometrial invasion.
- Measurement of depth of invasion is best performed on oblique axial post-contrast sequence—paralleling the pathologists’ histological assessment.

## Background

Endometrial cancer is the most common gynaecological cancer in developed countries [1, 2]. It predominantly affects post-menopausal women; however, younger cases can be seen in association with diabetes, obesity and Lynch syndrome, for example. Magnetic resonance imaging (MRI) has emerged as an informative imaging modality for local staging of endometrial cancer. This review aims to demonstrate the correlative radiological and pathological findings of endometrial carcinoma in various FIGO (International Federation of Gynecology and Obstetrics) stages as well as give examples of potential pitfalls where MR imaging is limited.

## Histopathology and clinical factors

**Fig. 1:**
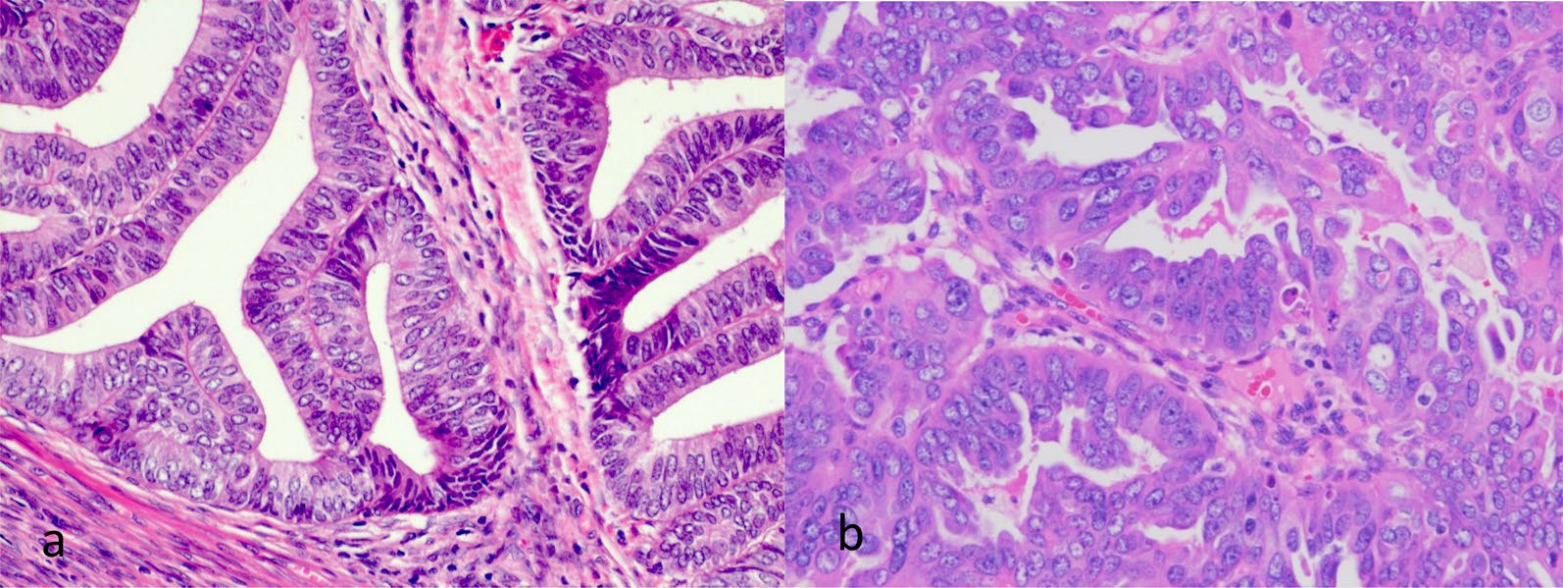
Histology images of Type 1 (**a**) and Type 2 (**b**) endometrial carcinomas. **a** shows a grade 1 endometrioid adenocarcinoma. There are well-formed glands with uniform appearing pencillate nuclei. **b** shows a serous adenocarcinoma—a Type 2 tumour with increased propensity for early extra-uterine spread. There is a more haphazard and disorderly glandular growth with pale enlarged nuclei and prominent nucleoli

Endometrial cancer can be broadly divided into two types: Type I and Type II (Fig. 1). The most common Type 1 endometrial cancer is endometrioid adenocarcinoma, which accounts for 75–80% of endometrial cancers [3] and often arises on a background of atypical hyperplasia. Type II is less oestrogen dependent and shows a more aggressive behaviour with tendency to deeper myometrial invasion. Serous, clear-cell and undifferentiated carcinoma makes up the common histological subtypes of the Type II cancer.

The specific histology subtype of the endometrial carcinoma can influence the prognosis as well as the pattern of extra-uterine spread. Endometrioid subtype tends to spread by direct extension and nodal disease initially. In contrast, serous and clear-cell endometrial carcinoma is more aggressive with increased lymphovascular invasion, peritoneal and extra-abdominal spread [3].

The prognosis of endometrial cancer is dependent on histological subtype, histological grade, lymphovascular invasion and FIGO staging [4, 5]. Tumours are at higher risk of lymph node metastases with higher grades or deeper myometrial invasion. In these patients, additional surgical staging with lymphadenectomy or sentinel node biopsy may be performed [5–7]. Lymphadenectomy does not confer survival advantage but allows nodal staging that identify patients that require further adjuvant therapy following surgery.

FIGO staging was last updated in 2009 [8] (Table 1). The majority of patients with endometrial carcinoma present at early stage. Over two-thirds (69%) of the women present with stage I disease at diagnosis, whilst other stages are less common (stage II 7%; stage III 10%; stage IV 7%; stage unknown 7%) (10). There has been a recent proposal for updating the staging. The proposal incorporates the histopathologic grade into the definition of stage 1 and eliminates the cervical involvement from the staging [9].

**Table 1:** FIGO (2009) classification of endometrial cancer

FIGO	Description
**Stage I**	Tumour confined to the corpus uteri
IA	No or less than half myometrial invasion
IB	Invasion equal to or more than half of the myometrium
**Stage II**	Tumour invades cervical stroma, but does not extend beyond the uterus*
**Stage III**	Local and/or regional spread of the tumour
IIIA	Tumour invades the serosa of the corpus uteri and/or adnexae (direct extension or metastasis)^#^
IIIB	Vaginal and/or parametrial involvement (direct extension or metastasis)^#^
IIIC	Metastases to pelvic and/or para-aortic lymph nodes^#^
IIIC1	Positive pelvic nodes
IIIC2	Positive para-aortic lymph nodes with or without positive pelvic lymph nodes
**Stage IV**	Tumour invades bladder and/or bowel mucosa, and/or distant metastases
IVA	Tumour invasion of bladder and/or bowel mucosa^§^
IVB	Distant metastases, including intra-abdominal metastases and/or inguinal lymph nodes

FIGO International Federation of Gynecology and Obstetrics

⁎Endocervical glandular involvement only should be considered as stage I and no longer as stage II

^#^Positive cytology has to be reported separately without changing the stage

^§^Bullous oedema in itself does not indicate mucosal invasion

## MRI technique

The MRI protocol for the imaging of endometrial carcinoma as well as the value of the individual sequences is detailed in Table 2. High-resolution T2 imaging helps to delineate the anatomy of the uterus and its relationship to adjacent organs. DWI is sensitive for tumour which shows diffusion restriction but has low spatial resolution. Dynamic contrast-enhanced (DCE) sequences with arterial phase allow visualisation of the normal early sub-endometrial myometrial enhancement. Contrast-enhanced sequence is the most reliable in differentiating between the tumour and the myometrium and optimal for the measurement of the depth of tumour invasion.

**Table 2:** MRI protocol and assessment

Sequence	Plane (s)	Slice thickness	Tumour characteristics and what to look for
T1W	Axial T1W	5 mm	- Endometrial cancer is isointense to myometrium on T1W - Haematometros is often seen post-biopsy and hyperintense
T2W	Sagittal T2W	4 mm	- Endometrial carcinoma has an intermediate signal on T2W - Best sequences for anatomical delineation - Assess depth of myometrial invasion and local staging
	Axial T2W	4 mm	
	Oblique axial T2W	3 mm	
DWI	Oblique axial DWI (b50, 500, 800)	4 mm	- Endometrial carcinoma shows diffusion restriction - Assess depth of myometrial invasion - Detection of small tumour deposits in the cervix, adnexa, or vagina - Detection of lymph nodes—however, does not distinguish between malignant and benign nodes
DCE and post-contrast T1W	Sagittal DCE T1W	3 mm	- Tumour enhancement is homogeneous, slower and less avid compared to the myometrium - Early enhanced imaging helps in detecting junctional zone (subendometrial myometrium) to exclude myometrial invasion - Loss of the normal rim of enhancement of the outer myometrium indicates serosal involvement - Assess depth of myometrial invasion - Assess presence of cervical or vaginal invasion
	3D Axial T1W FS with oblique axial reformat	1 mm	

T1W T1-weighted, T2W T2-weighted, DWI diffusion-weighted imaging, DCE dynamic contrast-enhanced sequence, FS fat-saturated

## Radiology–pathology correlation

Endometrial carcinoma is intermediate in signal intensity on T2-weighted imaging and hyperintense compared to the myometrium. The tumour is hypoenhancing relative to the background myometrium on the contrast-enhanced sequences—a useful feature in evaluating the depth of myometrium invasion. Normal endometrium, in contrast, shows delayed enhancement similar to the myometrium. The tumour has signal restriction on diffusion weighted imaging (DWI) due to the increased cellularity. DWI findings help to identify the tumour when small. The tumour can be quite variable in its growth pattern. It may have a large polypoidal intracavitary component, diffuse thickening of the endometrium or minimal intracavitary component with bulk of the tumour growing into the myometrium.

## Stage I

### Myometrial invasion

**Fig. 2:**
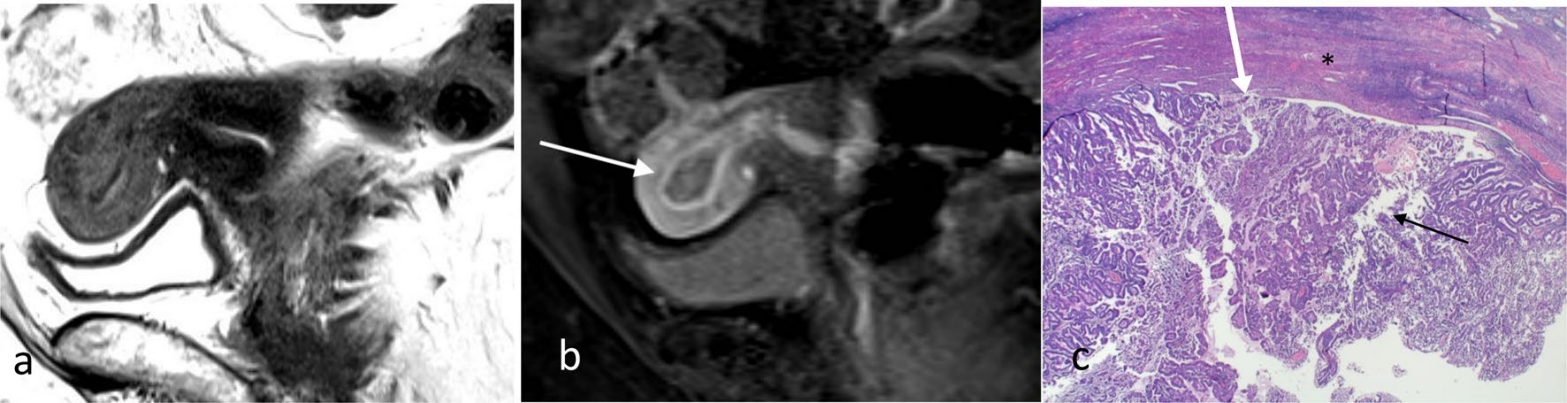
Stage 1A endometrioid endometrial carcinoma without myometrial invasion. Sagittal T2W image (**a**) shows the tumour is intermediate in signal (arrow). On early sagittal DCE image (**b**), there is smooth uninterrupted band of subendometrial junctional zone enhancement (arrow) which excludes myometrial invasion. Histology image (**c**) shows tumour (black arrow) confined to the endometrium and does not invade into the myometrium (*). There is preserved endometrium/myometrium junction (white arrow)

**Fig. 3:**
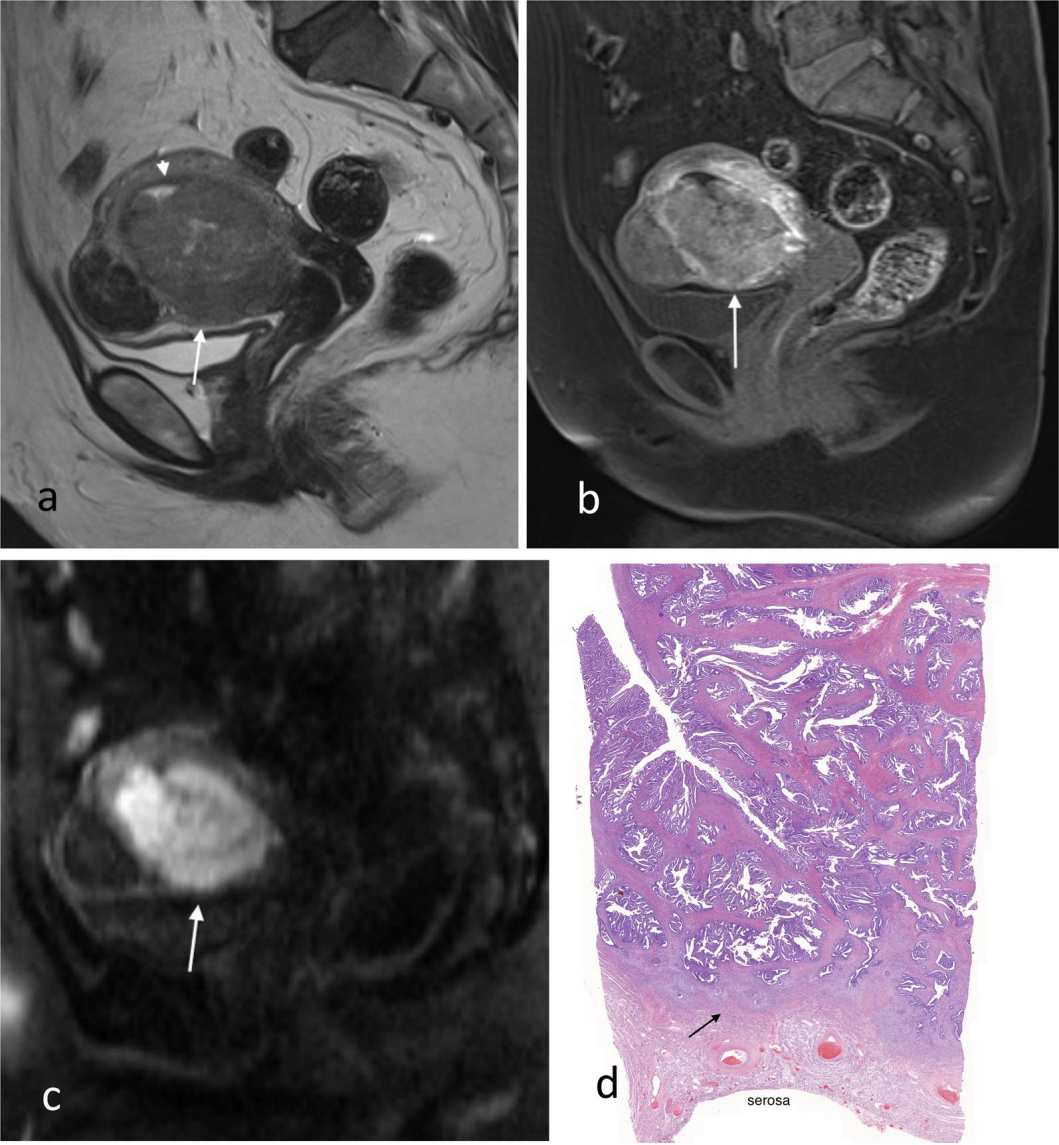
Stage IB endometrial carcinoma with extension to the outer half of the myometrium. Sagittal T2W image (**a**) shows the tumour (arrow) is bulky and extends into the outer half of the myometrium anteriorly. The outer uterine contour remains smooth. Junctional zone posteriorly is preserved as a thin T2 hypointense line (arrowhead). Sagittal early DCE images (**b**) show irregular peritumoural enhancement at the advancing front of the tumour (arrow) anteriorly, whilst the normal junctional zone posteriorly (arrowhead) has a smooth thin enhancement. Sagittal DW image (**c**) shows marked diffusion restriction of the tumour (arrow). Low-power histology (**d**) shows tumour extension to the outer half of the myometrium with preserved serosa and small amount of normal residual myometrium (arrow)

Most of the endometrial carcinoma presents at Stage 1. Stage IA and stage IB are differentiated by invasion greater than 50% of the myometrium (Figs. 2, 3). Whilst the volume of disease has been reported to be a potential prognostic factor, deep myometrial invasion is more important in prognosis indication and staging [10]. With deeper myometrial invasion, there is increased propensity for spread of cancer beyond the uterus, particularly to pelvic lymph nodes. Measurement of the depth of myometrial invasion is therefore critical in local staging of endometrial carcinoma.

**Fig. 4:**
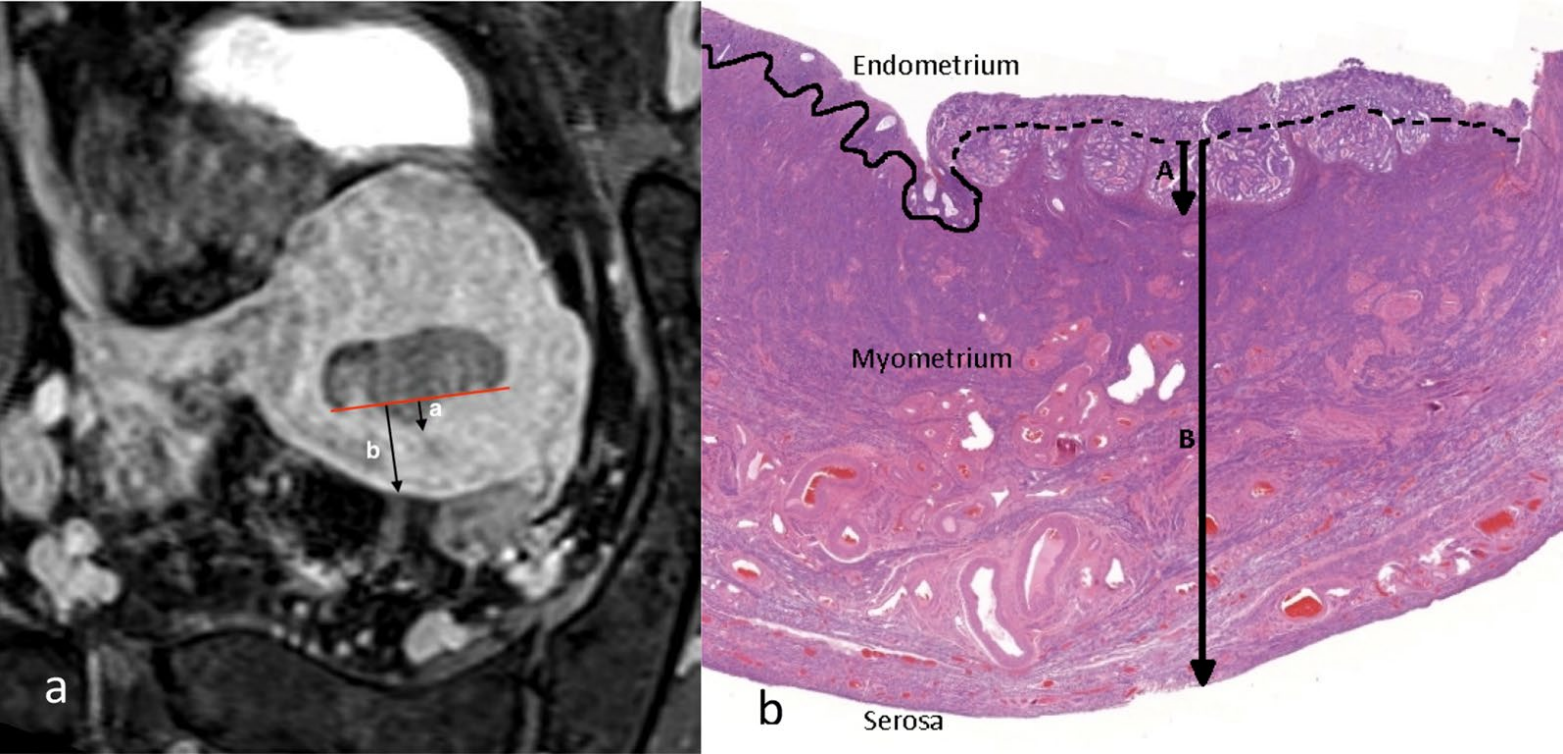
Radiological (**a**, oblique short-axis post-contrast T1W GRE fat suppressed image) and pathological (**b,** 0.4 × magnification) measurement of depth of myometrial invasion. (**a**) shows the radiological measurement of depth of invasion. The red line is an imaginary line estimating the endometrium/myometrium junction. Measurement (**a** arrow) shows the depth of the tumour into the myometrium beyond the junction. Measurement (b arrow) shows overall total thickness of the myometrium. The endometrium/myometrium junction can be irregular normally or due to distortion by the tumour. Measurement on histology is performed on low-power view with myoinvasion determined when tumour cell is seen interspersed with myocytes. The solid line delineates the normal endometrium from the myometrium. The dotted line is the estimated endometrium/myometrium junction at the site of the tumour and the basis for the measurement of myoinvasion

**Fig. 5:**
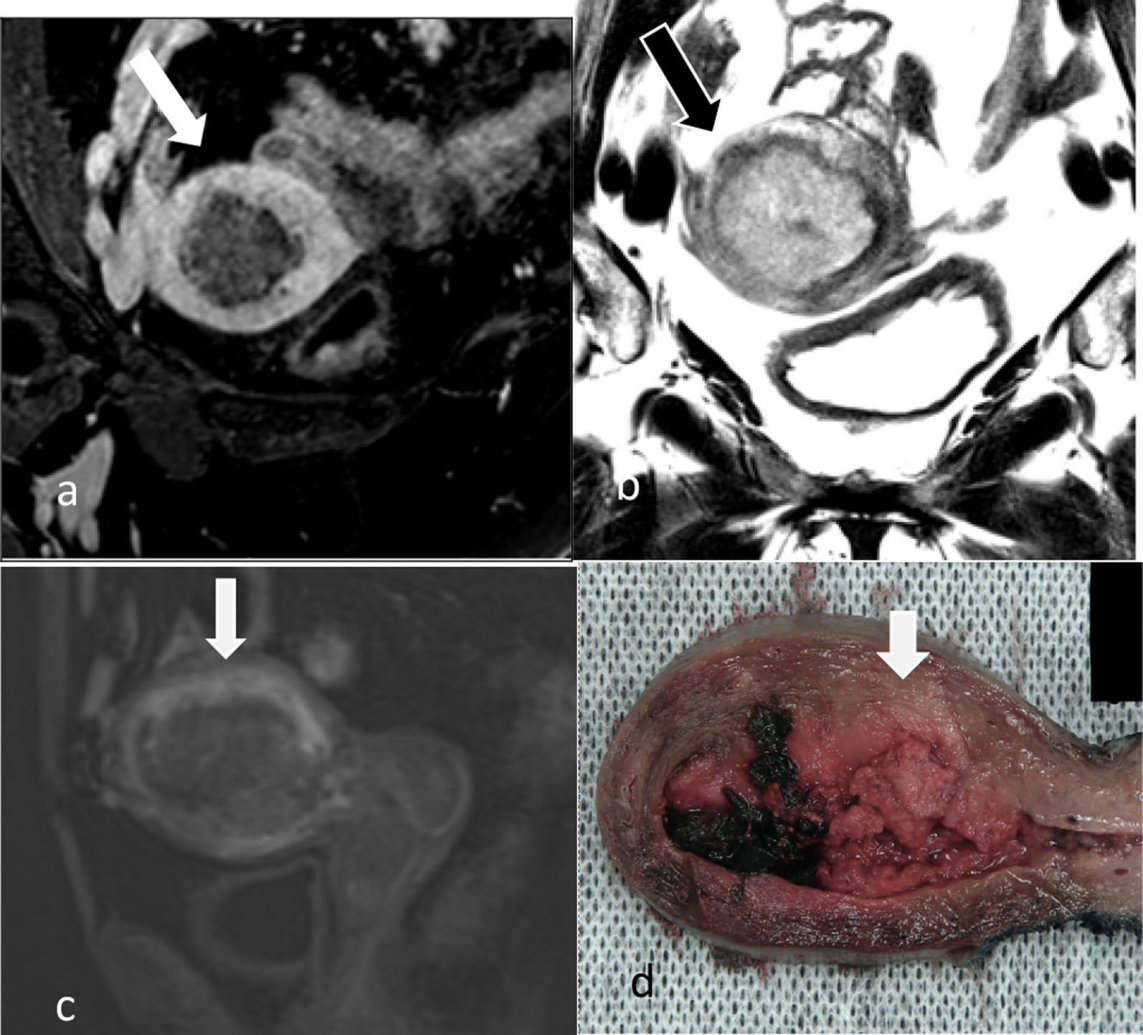
Oblique axial post-contrast 3D T1W GRE MR (**a**), oblique axial T2W MR (**b**), sagittal early phase DCE T1W MR (**c**) and macroscopic (**d**) images of a large endometrial carcinoma. There is a large exophytic intracavitary tumour with distension of the endometrial cavity. In this situation, it is difficult to estimate where the endometrium/myometrium interface should be and the depth of invasion. In this case, the tumour was extending into the outer half of the myometrium (stage 1b) at the posterior aspect on final pathology. The invasive front of the tumour (arrow) shows early peritumoural enhancement and has slight irregularity at the interface with the normal myometrium and blurring of junctional zone’s low T2 signal

**Fig. 6:**
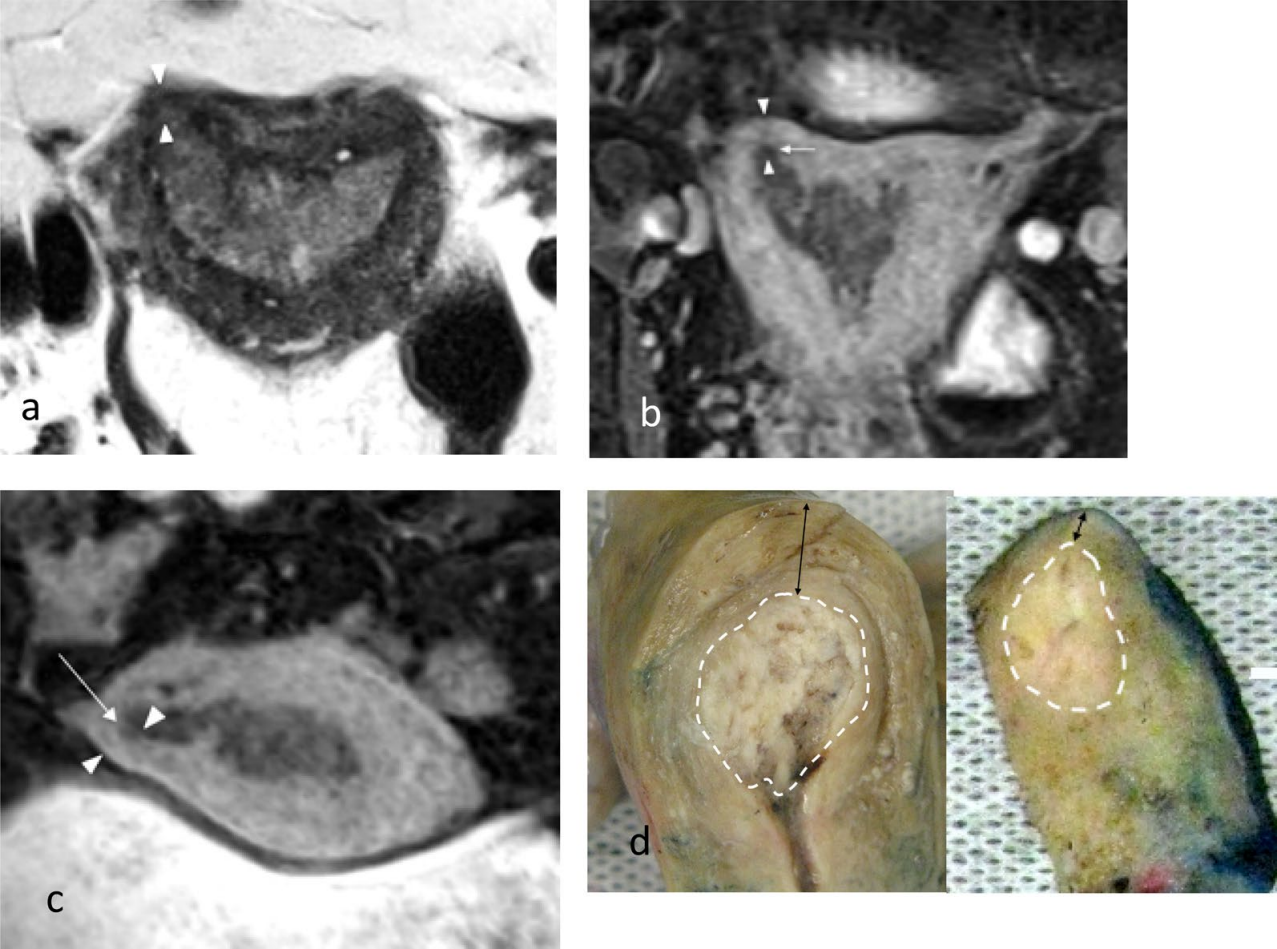
Tumour extending to the right cornua in a patient with stage 1a endometrial carcinoma. Axial T2W image (**a**), oblique coronal (**b**) and oblique axial (**c**) post-contrast 3D T1W GRE fat suppressed reformat images show the tumour involving the endometrial cavity and extending to the right cornu (arrow). There is normal thinning of the myometrium (arrowheads) at the cornu compared to the background myometrium. Midline sagittal and right cornu parasagittal macroscopic specimens (**d**) show the tumour (dotted white lines) and thinning of the myometrium (arrows) at the cornu. The background myometrial thinning has to be taken into account in the evaluation of the percentage of the depth of invasion to avoid overestimation

**Fig. 7:**
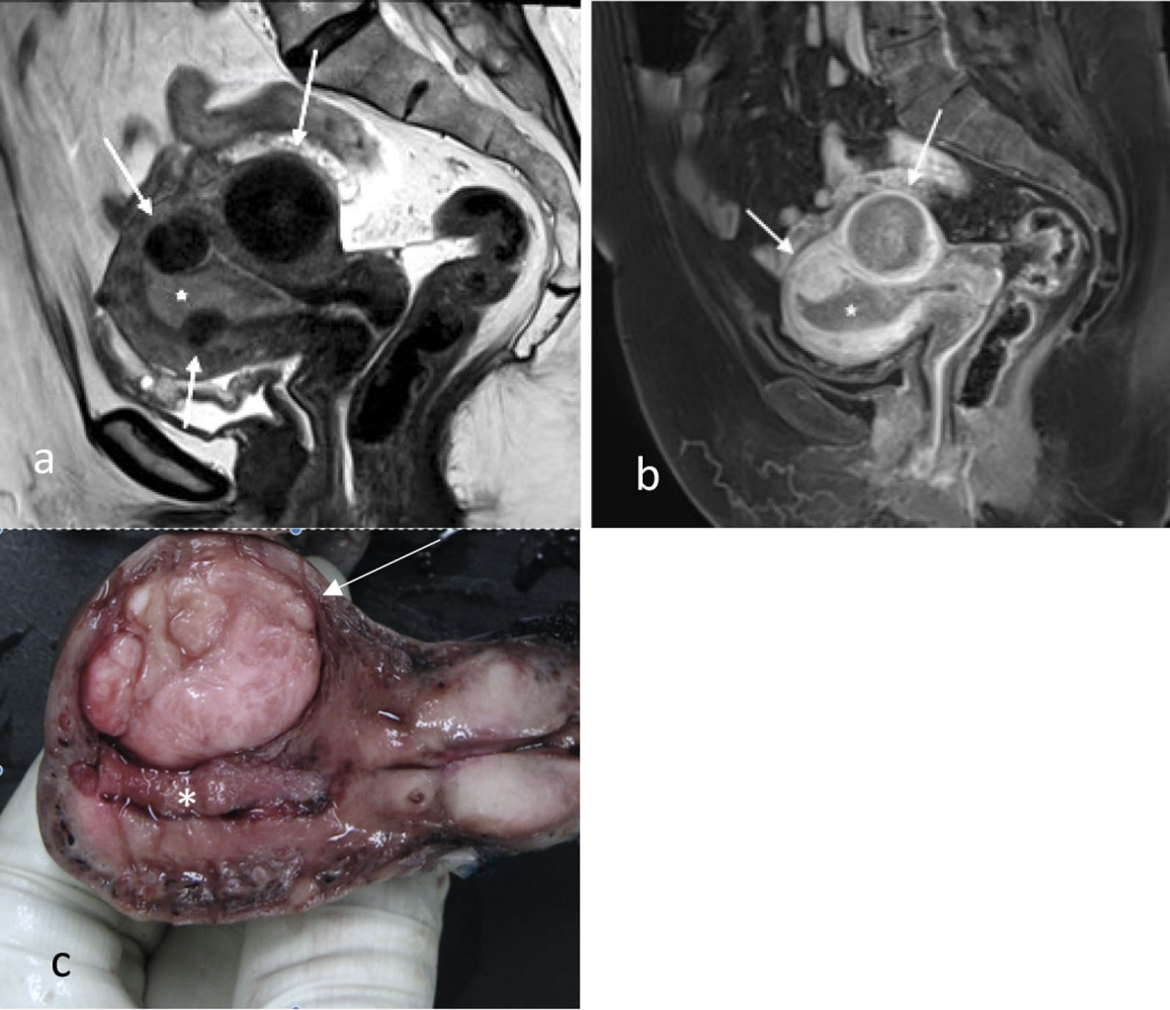
Sagittal T2W (**a**), sagittal post-contrast T1W (**b**) and macroscopic (**c**) images of stage 1a endometrial carcinoma (*) without myometrial invasion in a multi-fibroid uterus. The fibroids (arrows) are T2 hypointense and often hypoenhancing relative to the myometrium. The fibroids distort the endometrial–myometrial junction and complicate the assessment for myometrial invasion. The fundal fibroid shows similar enhancement relative to the myometrium

**Fig. 8:**
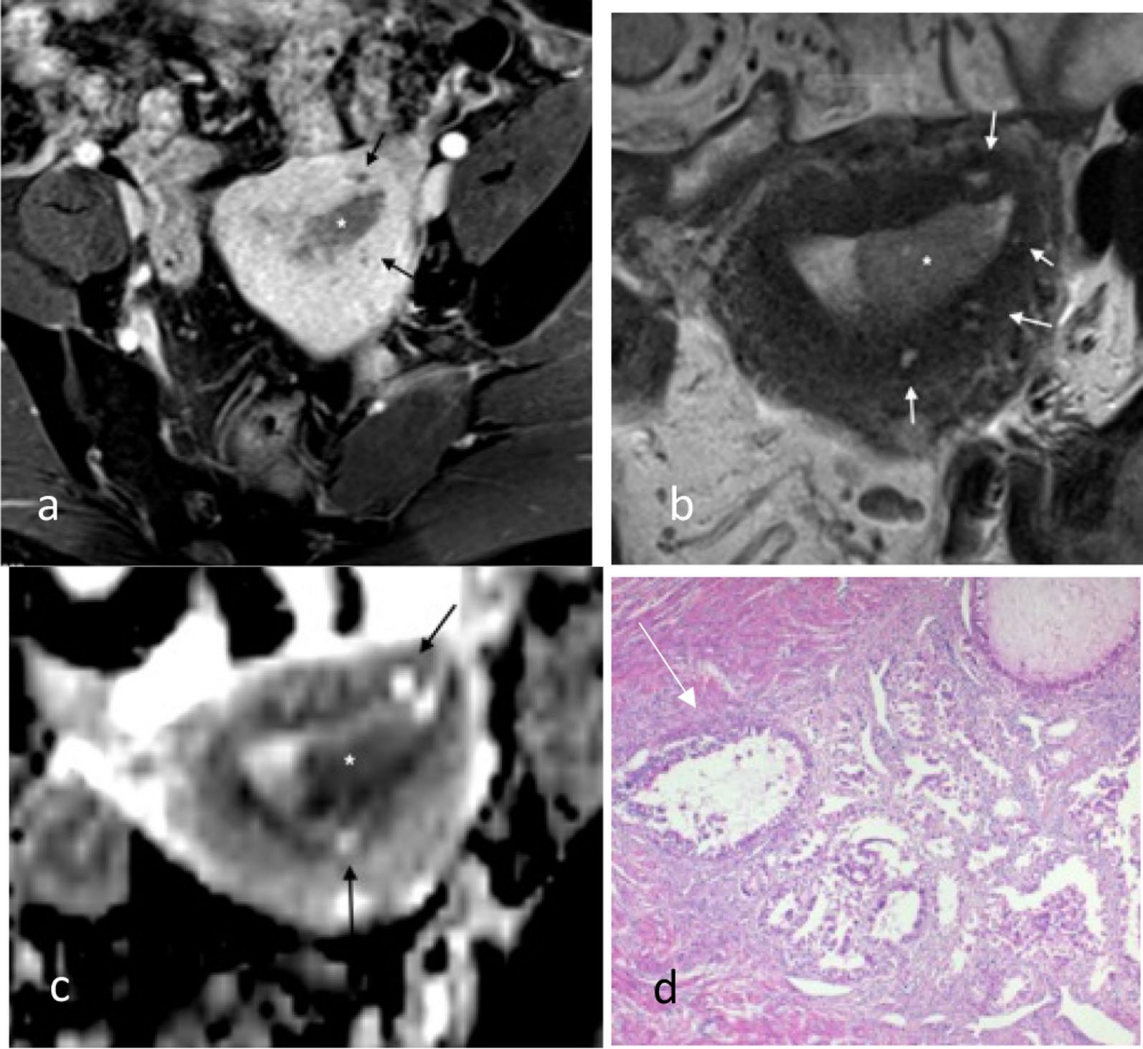
Endometrial carcinoma in a patient with adenomyosis. Axial post-contrast T1W GRE (**a**), axial T2W (**b**) and axial ADC map (**c**) images show the tumour (*) in the endometrial cavity. There is diffuse adenomyosis with background myometrial cysts (arrows). Myometrial cysts are hyperintense on T2 weighted imaging without significant diffusion restriction. Tumour can colonise adenomyosis and is not considered to be muscle invasive if confined to the adenomyosis. Histology (H&E stain, 4 × magnification, **d**) showed colonisation of areas of adenomyosis by complex crowded tumour cells (arrow), but this could not be preoperatively distinguished from normal adenomyosis on imaging

**Fig. 9:**
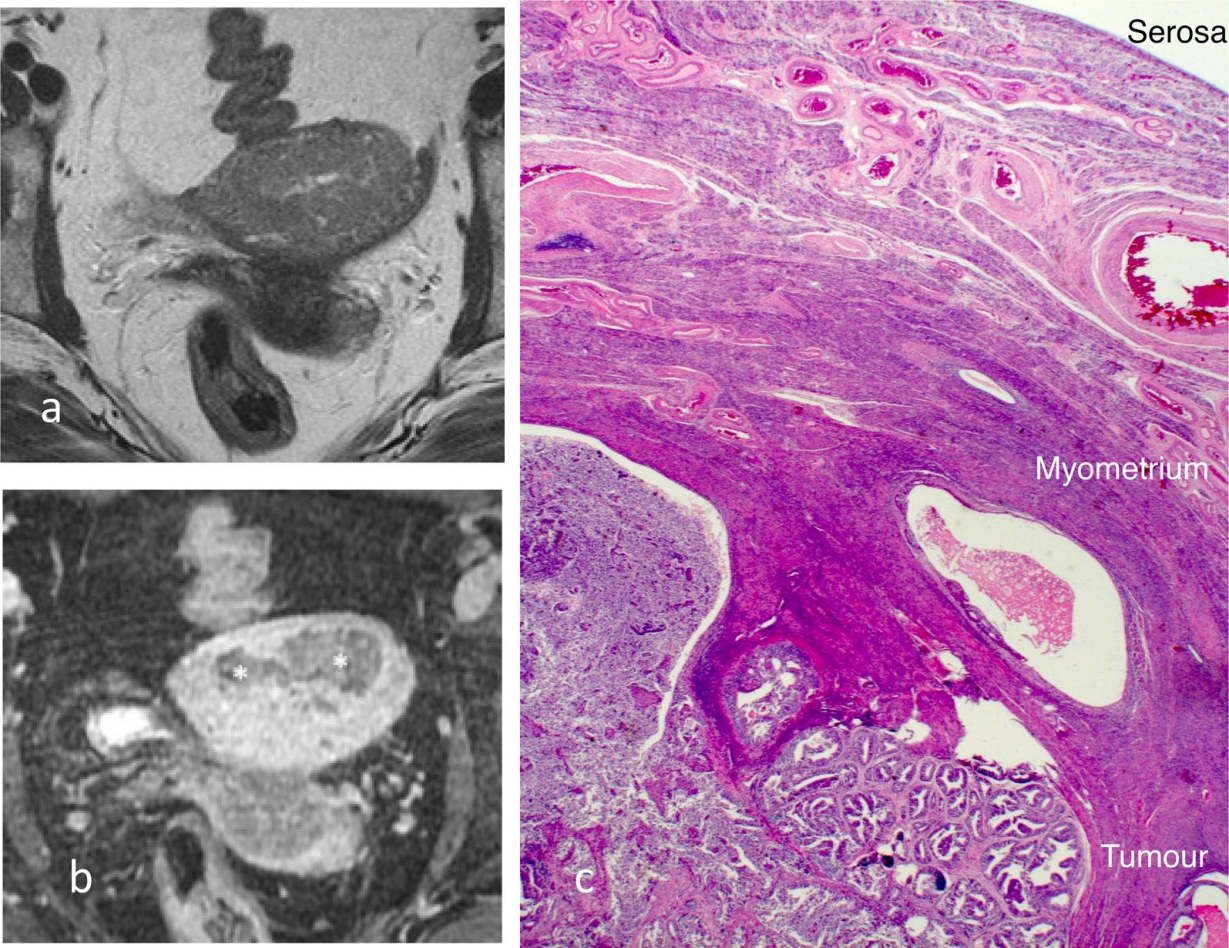
Endometrial carcinoma with T2 signal isointense to myometrium. On axial T2W MR image (**a**), the tumour has the same signal intensity to the adjacent myometrium. It is difficult to appreciate the exact border on T2W MR imaging. On the post-contrast T1W imaging (**b**), there is better tumour (*) to myometrium contrast. Microscopic image (**c**) confirms tumour invasion into the myometrium

**Fig. 10:**
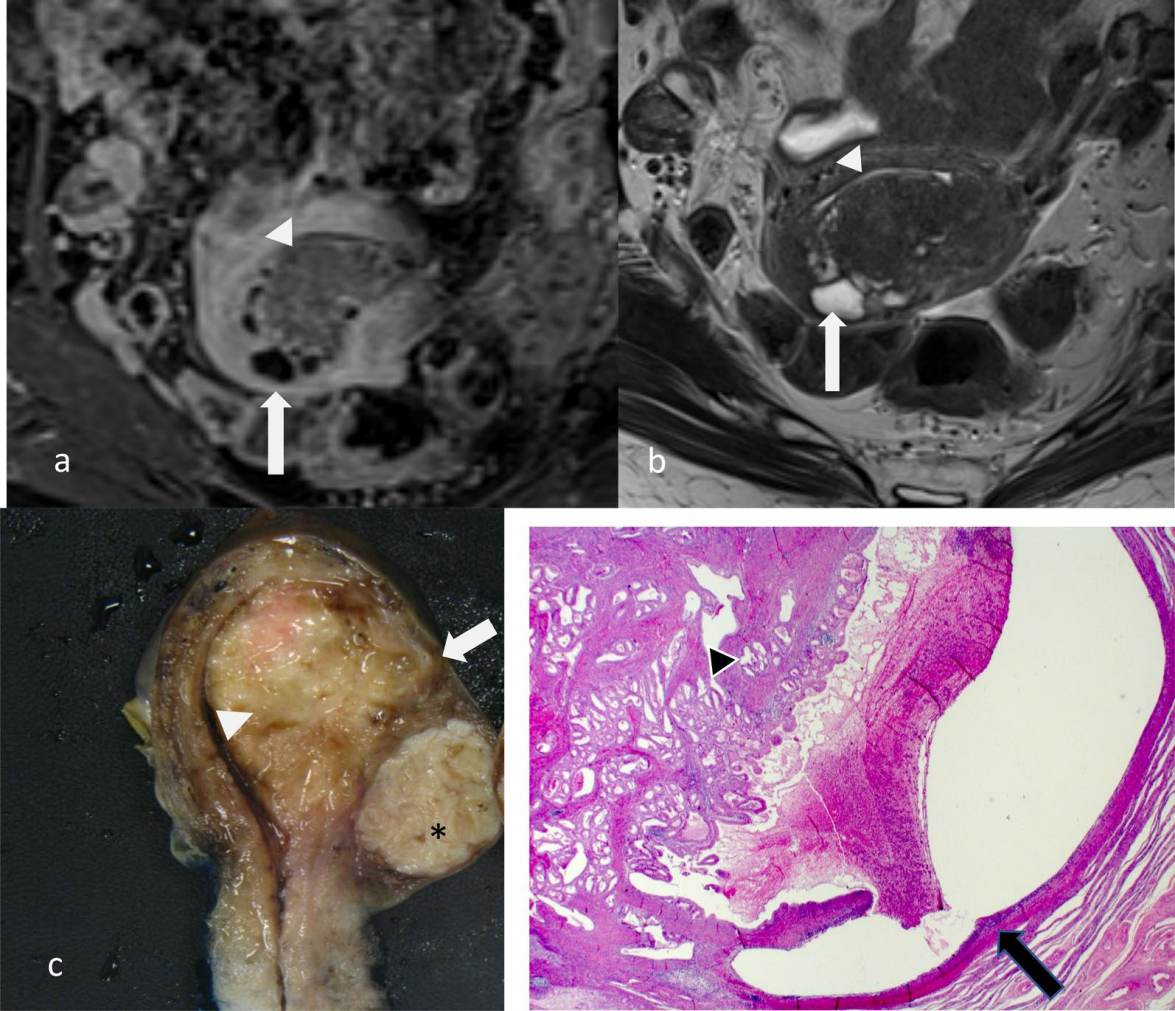
Endometrial carcinoma with mucinous differentiation. On the oblique axial post-contrast T1W (**a**) and the T2W (**b**) MR images, the tumour is accompanied by cystic spaces that are T2 hyperintense without enhancement (arrows). These cystic areas on pathology (**c**) correlate with mucin pools (arrow) associated with the tumour (arrowheads) that extend into the outer half of the myometrium. There is a fibroid in the lower aspect of the uterus (*). Microscopic image (**d**) shows endometrioid carcinoma (arrowhead) with mucinous differentiation and large space adjacent (arrow) containing acellular proteinaceous material consistent with mucin

The measurement on histology is based on low-field microscopy and is defined as tumour invasion into myometrial smooth muscle, relative to the estimated depth of the entire myometrium at that point (Fig. 4) [11, 12]. The radiological measurement is best performed on short-axis oblique post-contrast sequence through the uterus [4–6, 13, 14]. The measurement, however, can be difficult on both imaging and pathology, and the potential pitfalls are listed in Table 3 (Figs. 3, 5, 6, 7, 8, 9, 10)[15].

**Table 3:** Radiological assessment of myometrial invasion

Scenario	Difficulty	Tips
Large tumour volume (Fig. 5)	- Difficulty in identifying the endometrium/myometrium junction - Thinning of the myometrium because of tumoural distension	- Symmetry and smooth interface favour endometrium confined disease (stage IA) - Smooth uninterrupted band of subendometrial enhancement on early DCE excludes myometrial invasion
Peritumoural enhancement (Figs. 3, 5)	- Some tumours exhibit peritumoural enhancement which may be difficult to distinguish from junctional zone enhancement	- Correlate with T2W sequences. Normal junctional zone has low T2W signal compared to normal myometrium
Tumour located in the cornua (Fig. 6)	- Thin overlying myometrium at cornua makes it difficult to estimate percentage of invasion	- Compare to the opposite cornua and be aware of the that there is normal thinning of the myometrium at the cornua - Ensure correct angulation on the oblique reformats of the contrast-enhanced T1 3D volume sequence to enable comparison
Tumour in multi-fibroid uterus (Fig. 7)	- Distortion of both the myometrial–endometrial junction and the depth of the myometrium	- Look at contrast-enhanced sequences and DWI to delineate the tumour from fibroids
Tumour in uterus with adenomyosis (Fig. 8)	- Difficulty assessing myometrial invasion versus adenomyosis versus tumour colonisation in adenomyosis	- Look at DWI as adenomyosis usually does not show diffusion restriction - Tumour confined to adenomyosis is not considered to be myoinvasive
Tumour is isointense to myometrium on T2W (Fig. 9)	- Difficulty in delineating tumour margins and identifying extent of myometrial or serosal invasion	- Post-contrast sequences are best for delineating tumour depth and avoids overestimation
Tumour with mucinous cystic components (Fig. 10)	Tumours may have mucinous differentiation and develop mucin pools that appear cystic on MRI. These mucin pools when present should be included in the depth evaluation	- Awareness of the possible histology of mucinous differentiation in endometrial carcinoma helps radiologists to be prepared for the variation in the appearance of the tumour

The endometrium/myometrium interface is an important landmark, but it can be irregular or distorted due to fibroids or background adenomyosis. Identifying normal physiological enhancement of the subendometrial myometrium (junction zone) on the early DCE sequences is important to exclude myometrial invasion. However, it is not always present due to either timing of the scan or tumour involvement. It can also occasionally be mimicked by the early enhancement at the pushing front of the tumour. The latter tends to be irregular, thick and associated with large tumours.

Large tumour volume may also distend the endometrial cavity. The intracavitary component of the large tumour may be erroneously included as part of the tumour depth into the myometrium leading to overestimation of the depth of invasion.

Atypical appearance of the tumour can be seen particularly in relation to T2 signal. Occasionally, tumour may be isointense to the myometrium or contain T2 hyperintense mucinous components. In those cases, the contrast-enhanced sequences are more reliable in marking out the extent of the tumour.

## Stage II

### Cervical stromal invasion

**Fig. 11:**
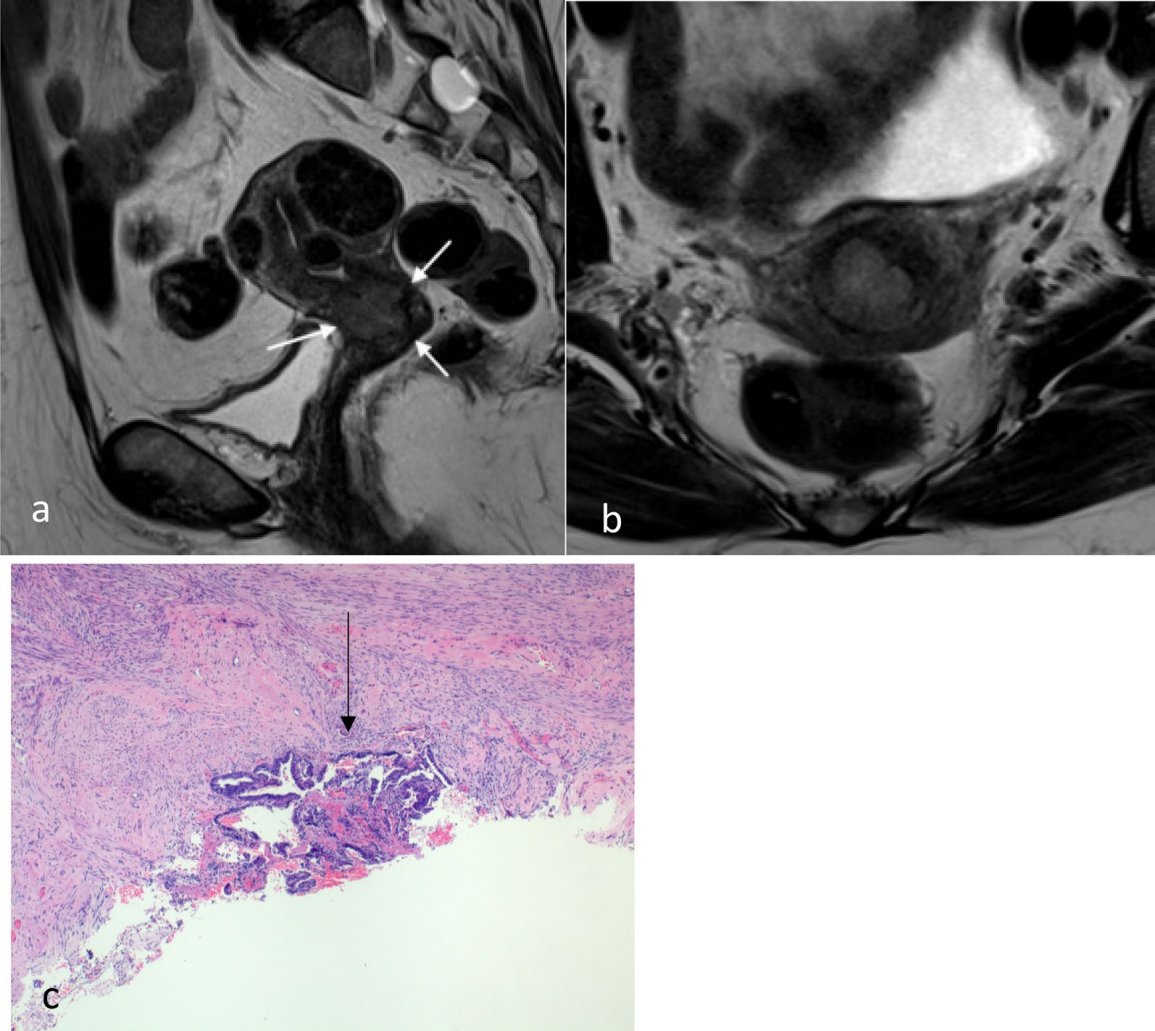
Stage II endometrial carcinoma with cervical stroma involvement. On sagittal (**a**) and axial (**b**) T2W MR imaging, there is intermediate T2 signal tumour (arrows) replacing the low signal dense cervical stromal tissue. In this case, the tumour involving the cervix is from a drop metastasis from a smaller primary in the endometrial cavity (not shown). Pathology (**c**) determines cervical invasion based on identification of the haphazardly arranged tumour cells (arrow) invading beyond the confines of cervical mucosa with surrounding stromal reaction

The invasion of the cervical stroma denotes stage II (Fig. 11). Cervical stroma is differentiated from the myometrium based on the type of supporting stroma on histology. Whilst the uterus has smooth muscle bundles, the cervix has dense fibrocollagenous tissue. On MRI, the cervical stroma has lower T2 signal reflective of the dense stroma. Cervical stromal invasion is seen on delayed contrast-enhanced images as disruption of normal cervical enhancement by hypoenhancing tumour. Tumour involvement of only the surface mucosa or glandular epithelium of the cervix without stromal involvement remains stage I. Tumour extending into the endocervical canal without actual disruption of the low T2 signal of the cervical stroma should not be considered cervical stromal invasion. Cervical stromal involvement can be due to contiguous involvement or may be distant from the tumour as a so-called drop metastasis.

## Stage IIIA

### Serosal involvement

**Fig. 12:**
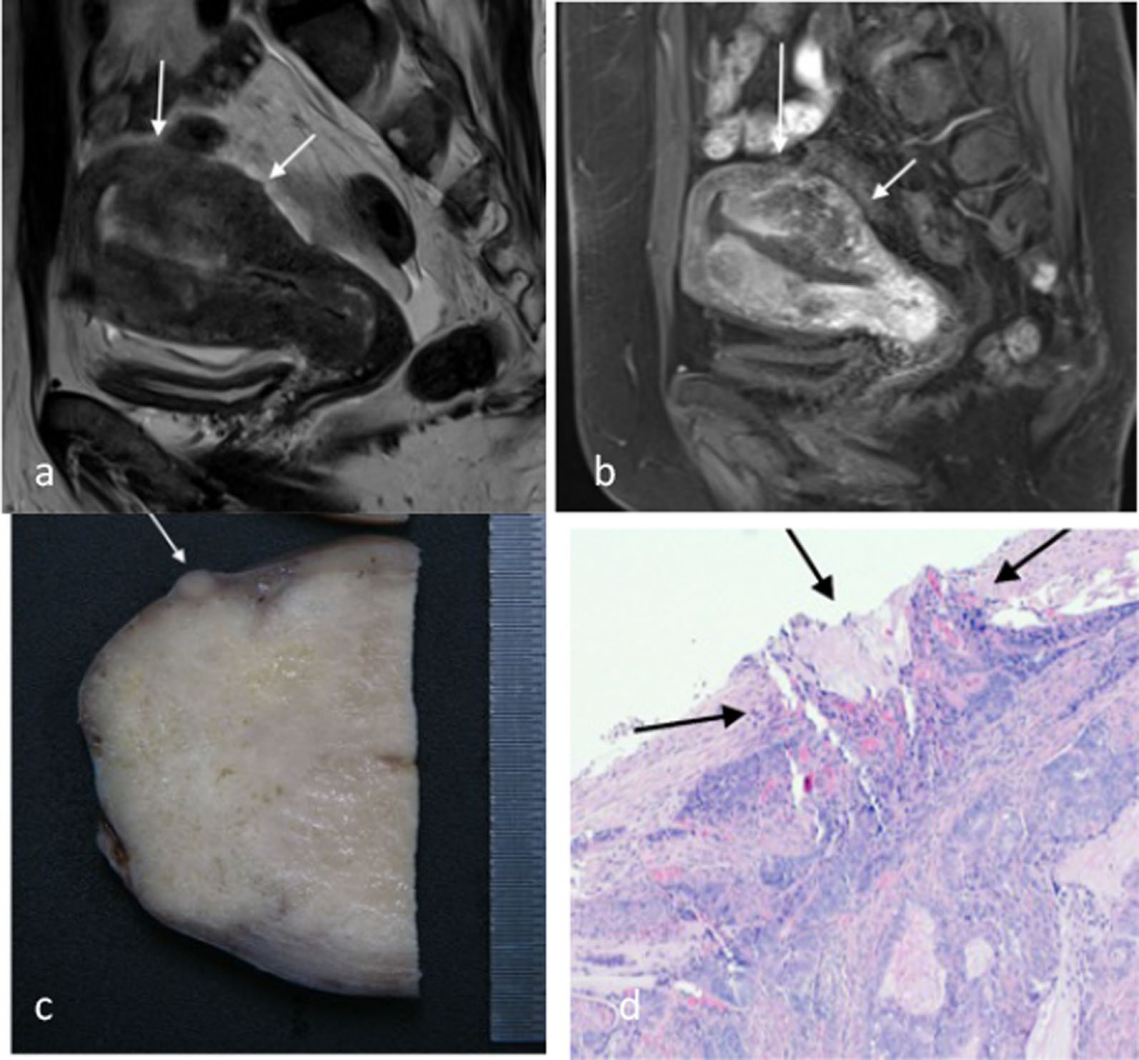
Stage IIIA endometrial carcinoma with serosal involvement. There is irregularity at the posterior uterine outer surface (arrows) on sagittal T2W (**a**) and sagittal DCE T1 MR imaging (**b**) in addition to full thickness tumour signal involvement of the myometrium. Pathological examples of serosal involvement are shown in **c**, **d**. Macroscopically, there is a homogeneous tan nodule at the serosal surface (arrow, **c**). Microscopically, this correlates with infiltrating hyperchromatic tumour cells extending through the outer myometrium and erupting through the serosal tissue (arrow, **d**)

Involvement of the serosa (stage IIIA) on MRI is characterised by full thickness tumour signal replacement of the myometrium with irregularity at the uterine outer surface (Fig. 12). This correlates with histological finding of extension of tumour beyond the myometrium and into the serosal layer. This can be seen on microscopic examination and sometimes on macroscopic examination as well (Fig. 12).

### Ovarian involvement

**Fig. 13:**
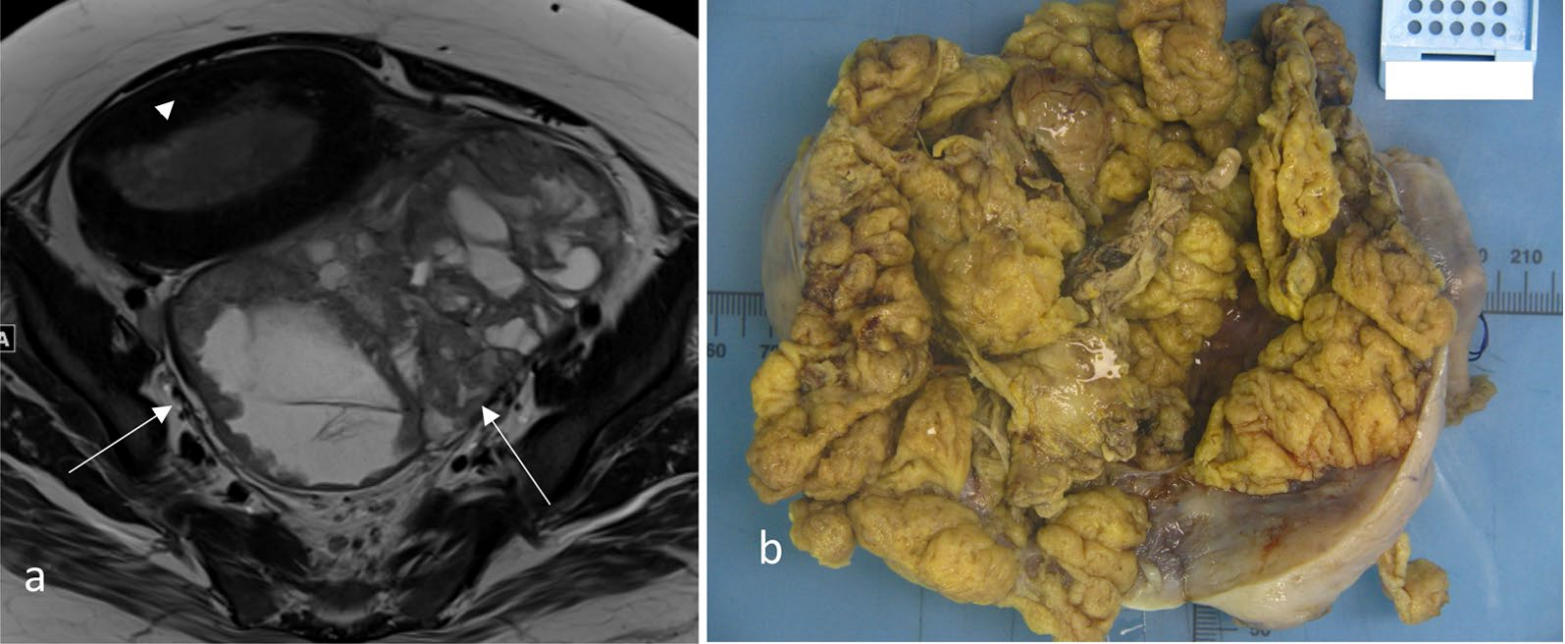
Stage IIIA endometrial carcinoma. Axial T2W MR image (**a**) shows a left ovarian metastasis (arrow) separate from the primary tumour (arrowhead). Macroscopic pathology specimen (**b**) shows open ovary with internal friable and tan solid components. Ovarian involvement can be due to direct invasion, haematogenous spread or transcoelomic spread resulting in serosal tumour deposits

**Fig. 14:**
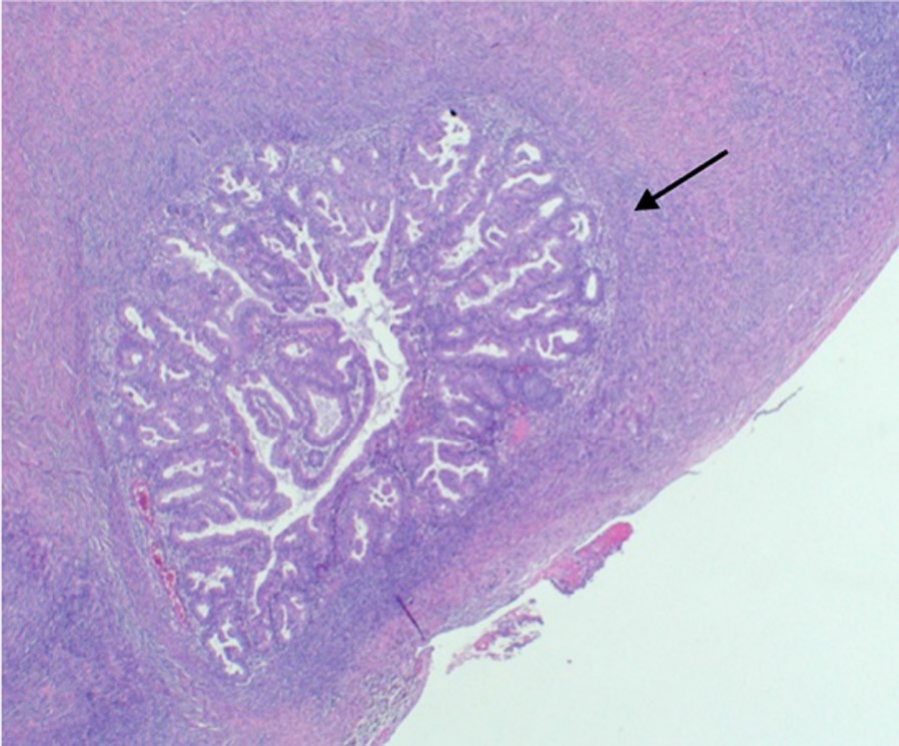
Serosal deposit in ovarian parenchyma. Histology image (H&E stain, 2 × magnification) shows a small serosal deposit in the ovarian parenchyma. This was not visible on MRI. The small volume of disease may account for the lower sensitivity of MRI for ovarian metastases in endometrial carcinoma

Ovarian involvement (stage IIIA) can occur with contiguous spread of the primary tumour with engulfment of the normal ovarian tissue (Fig. 13). It can also occur as part of the peritoneal spread process on the ovarian serosa (Figs. 13, 14). It is important to be aware that synchronous ovarian tumour either benign or malignant can occur in conjunction with endometrial carcinoma. Not all ovarian lesions are due to metastases. A primary endometrial and secondary ovarian metastasis are more likely when there is a large volume endometrial tumour, high tumour grade and/or deep myometrial invasion in combination with a small ovarian lesion or when there is bilateral ovarian involvement [16].

## Stage IIIB

### Vaginal involvement

**Fig. 15:**
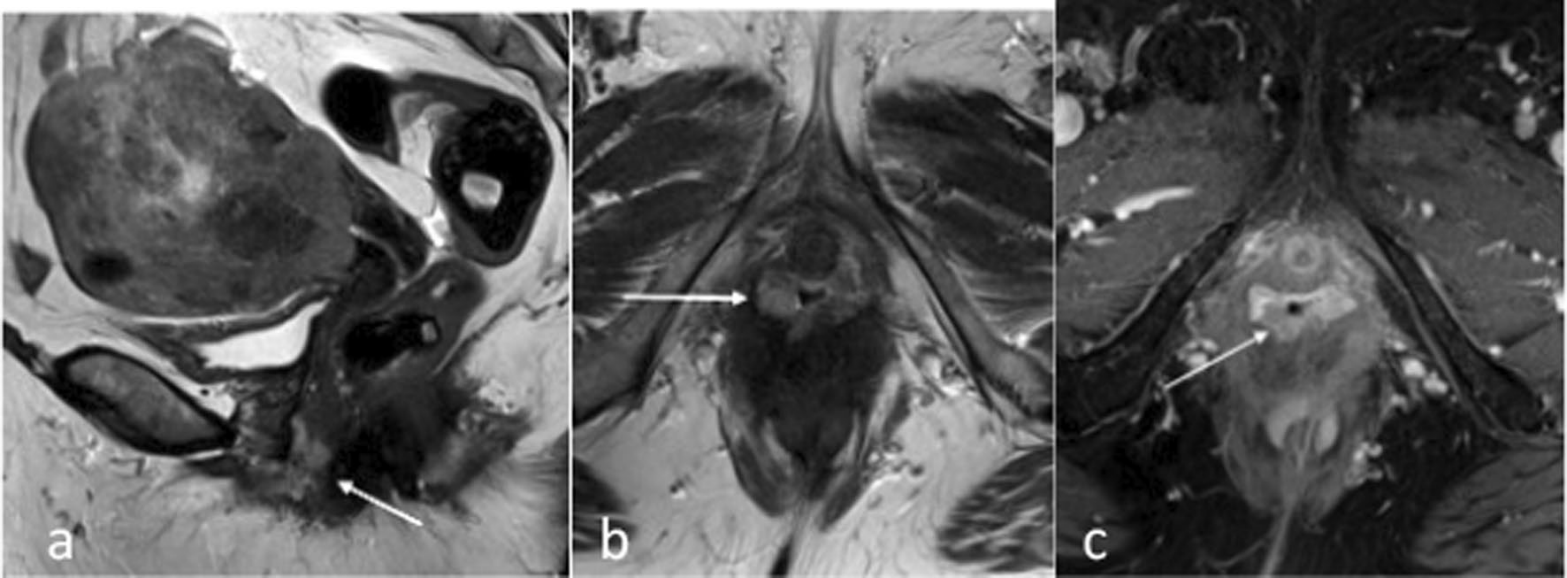
Stage IIIB endometrial carcinoma with vaginal involvement. There is a large tumour with extension through the myometrium into the serosa. On the sagittal (**a**) and axial (**b**) T2W MR images, there is a nodule (arrow) with intermediate T2 signal caudally at the level of the vagina on the right, in keeping with a drop metastasis. On axial post-contrast T1W image (**c**), the drop metastasis is an hypoenhancing lesion (arrow) compared to the hyperenhancing vaginal mucosa and muscular layer. This was diagnosed with biopsies confirming endometrioid carcinoma

Vaginal involvement at time of initial diagnosis is rare. Vaginal involvement commonly is due to drop metastasis (Fig. 15) and is separate from the primary tumour. Direct contiguous involvement of the vagina is very uncommon. Involvement of the vagina (stage IIIB) is readily apparent on clinical examination and can be difficult to identify on MRI when the drop metastasis is small, and the vaginal vault is collapsed. Review of the entire genital tract down to the level of the vaginal introitus on the MRI is helpful to ensure that larger drop metastases are not missed.

### Parametrial involvement

**Fig. 16:**
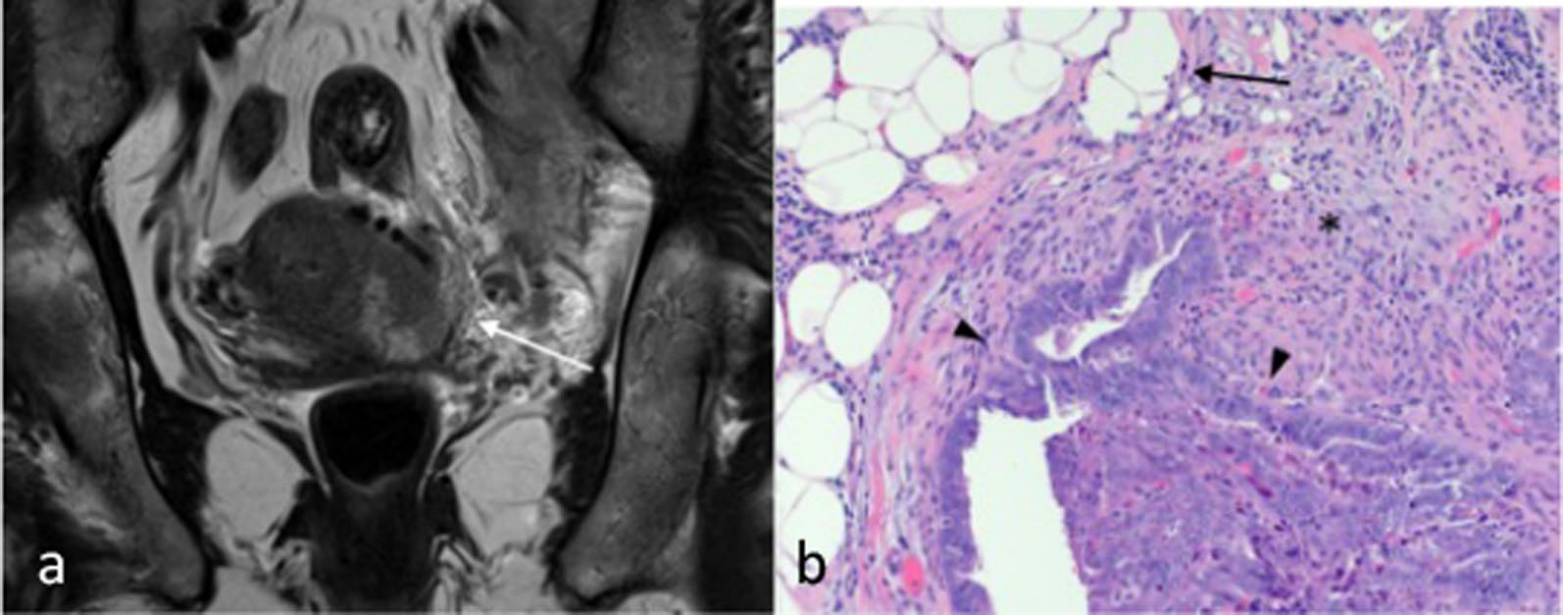
Stage IIIB endometrial carcinoma with parametrial invasion. Coronal T2W MR image (**a**) shows irregular intermediate T2 tumour signal replacing the entirety of the cervix and extending into the left parametrial fat in keeping with parametrial invasion (arrow). Parametrial invasion occurs from contiguous spread via the cervix. On histological image (× 10 magnification) (**b**), this correlates with the presence of tumour cells (arrowheads) infiltrating into adjacent adipose tissue (arrow) with a desmoplastic stromal response (*)

Radiologically, parametrial involvement occurs in the context of tumour involvement of the cervix. The involvement of the parametrium can be identified on MRI as loss of the full thickness low T2 cervical stroma signal with irregular interface at the adjacent parametrial fat or frank tumour extension into the fat (Fig. 16). On histology, this correlates with tumour cells with surrounding adipose tissue either by direct (continuous) or metastatic (discontinuous) spread [12].

### Lymph nodes

**Fig. 17:**
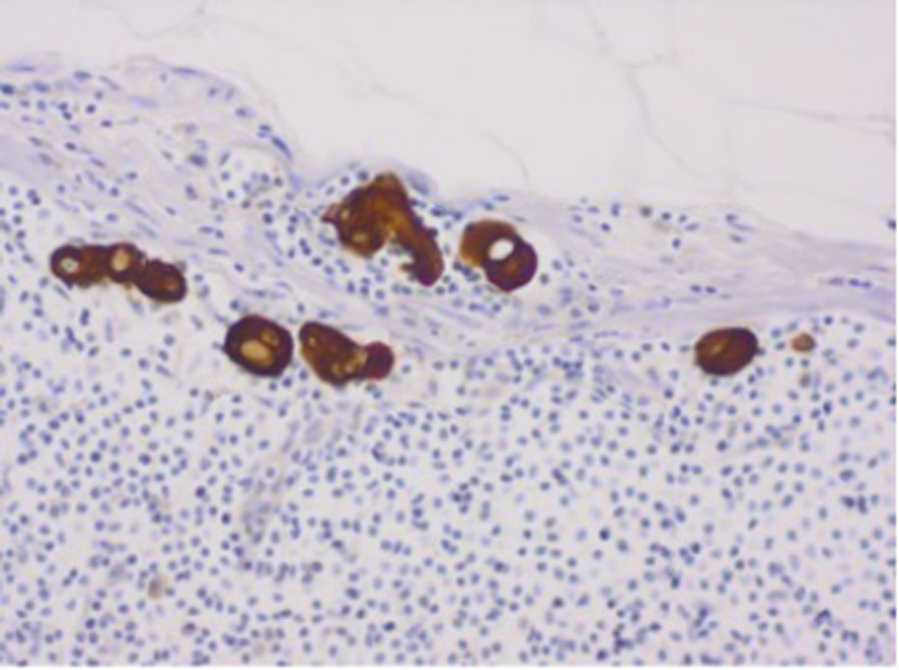
Isolated tumour cells less than 0.2 mm diameter in a lymph node detected on AE1/AE3 cytokeratin immunohistochemistry stain (× 20 magnification). Such low-volume disease in nodal spread may account for MRI’s limited sensitivity for lymph node involvement

The evaluation of the para-aortic nodes is easier on CT unless additional large field of view sequences are obtained through the abdomen. The evaluation of the pelvic nodes on MRI is still based on the short-axis dimension with a short-axis diameter cut-off of > 8 mm for pelvic lymph nodes and > 10 mm for para-aortic lymph nodes [6, 13]. Whilst occasionally, heterogeneity with tumour signal may be helpful to indicate involvement, this is not sensitive or specific [4–6, 13]. Benign and malignant nodes both show diffusion restriction. Tumour deposits may be microscopic, i.e. < 2 mm (N1mi) with no extra-capsular extension (Fig. 17). Inguinal nodes are considered to be stage IV with the other pelvic nodes considered to be stage IIIC.

## Stage IV

### Pelvic organ involvement

**Fig. 18:**
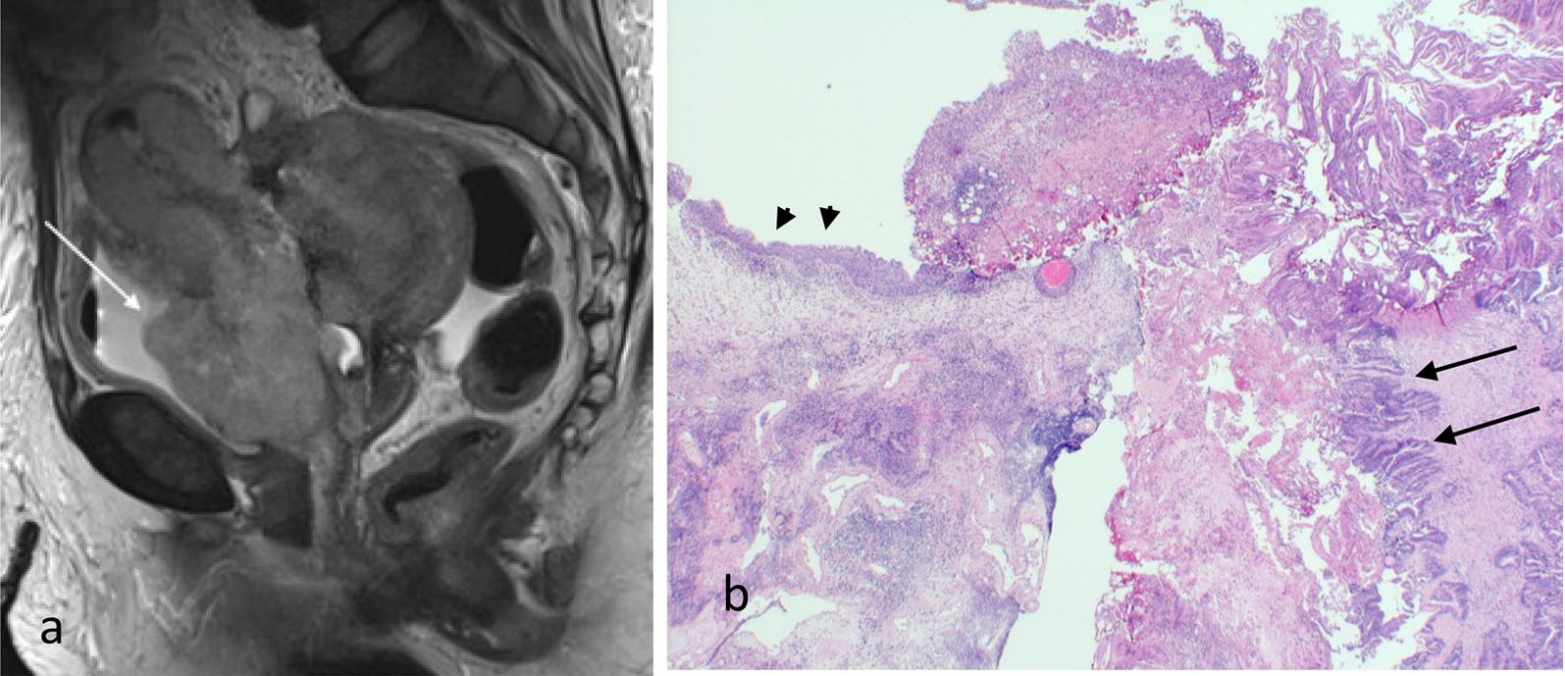
Stage IVA endometrial carcinoma. Sagittal T2W MR images (**a**) show direct tumour extension into the bladder mucosa (arrow). The air anteriorly in the bladder is due to a fistula. Chips of a bladder tumour (**b**, H&E stain, × 4 magnification) initially thought to be primary urothelial tumour show fragments of necrotic endometrioid adenocarcinoma (arrow) confirmed on immunohistochemistry. The normal bladder transitional epithelium is indicated by the arrowheads

Whilst rare, endometrial cancer can invade adjacent pelvic organs such as bladder and bowel (Fig. 18). This would occur in late presentation of the disease. Such pattern of advance pelvic invasion is more commonly seen with cervical carcinoma.

The high-resolution T2 sequences are optimal for the assessing the relationship of the tumour to the adjacent organs. The local invasion on MRI shows tumour signal that disrupts the adjacent organs such as bowel or bladder. Bladder involvement requires extension to the bladder urothelium. Isolated detrusor muscle involvement is not considered to be stage IV disease. Often, involvement of the urinary bladder detrusor muscle leads to an oedematous mucosa (bullous oedema) in the bladder which can be seen on MRI or cystoscopy. Protuberant tumour in the bladder lumen with acute angle relative to the bladder inner layer would be highly concerning for urothelial involvement. The finding of a utero-vesical fistula also invariably infers the presence of bladder involvement.

## Conclusion

Endometrial carcinoma is the most common gynaecological cancer. Whilst most cases are low grade with low-volume disease, it can also present with higher grade and more advanced disease with higher risk of nodal spread. MRI can assist in the local staging of the endometrial carcinoma preoperatively and guide the surgical and management decisions. MRI can assess the depth of myometrial invasion and distinguish between stage 1A and stage 1B disease. Its accuracy can, however, be affected by factors such as fibroids, adenomyosis, atrophic myometrium, bulky tumour, indistinct or variegated endometrium/myometrium junction and atypical tumour appearance on MRI. Understanding these potential pitfalls would help the reporter to improve the accuracy in detection of deep myometrial invasion. MRI with its higher tissue contrast resolution also has a role in identifying the extra-uterine pelvic disease. The extra-uterine disease can be due to different patterns of spread including contiguous, drop metastases, transcoelomic and lymphatic nodal spread. We present in this paper the radiology–pathology correlation at the different FIGO stages to help reporting radiologists to have a better understanding of the local spread of endometrial carcinoma and its imaging appearance.

## Data Availability

Not applicable.

## Abbreviations

DCE: Dynamic contrast enhanced
FIGO: The International Federation of Gynecology and Obstetrics
MRI: Magnetic resonance imaging
